# Challenges in the Long-Term Management of Stent-Related Complications: A Case of Recurrent Stent Thrombosis and Restenosis

**DOI:** 10.7759/cureus.88504

**Published:** 2025-07-22

**Authors:** Amine El Houari, Wassim Beladel, Enver Hilic, Sylvain Chanseaume, Mohamed El Minaoui

**Affiliations:** 1 Department of Cardiology, University Hospital Centre Souss Massa, Faculty of Medicine and Pharmacy, Ibn Zohr University, Agadir, MAR; 2 Department of Cardiology, Montluçon Hospital, Montluçon, FRA

**Keywords:** case report, drug eluting stents, in-stent restenosis, percutaneous coronary intervention, recurrent stent failure, stent thrombosis

## Abstract

Coronary stenting has substantially enhanced revascularization outcomes; however, in-stent restenosis (ISR) and stent thrombosis (ST) remain significant complications associated with increased morbidity and mortality. We present the case of a 63-year-old male with a history of ischemic heart disease who underwent percutaneous coronary intervention (PCI) for proximal left anterior descending (LAD) and right coronary artery (RCA) stenoses. Over a five-and-a-half-year follow-up period, he experienced two distinct episodes of ST, one occurring early and the other classified as very late, as well as a single occurrence of ISR, each requiring targeted interventional management. This case highlights the complex interplay of mechanisms underlying recurrent stent failure, emphasizing the need for personalized management and ongoing refinement of stent technologies and adjunctive therapies to improve long-term clinical outcomes.

## Introduction

Coronary stenting has revolutionized revascularization strategies, offering excellent procedural and clinical outcomes across a wide range of clinical scenarios. Despite its proven short- and long-term benefits, two major complications, in-stent restenosis (ISR) and stent thrombosis (ST), continue to limit its efficacy.

ST is a rare but devastating complication of percutaneous coronary intervention (PCI). Historically, the incidence of ST was reported as high as 16% during the early era of stent deployment [1]. Although modern practices have reduced the incidence dramatically, it remains a clinical concern. Current data indicate that the incidence of ST is approximately 0.7% within the first year, declining to 0.2-0.6% thereafter [1]. The risk is lower for elective PCI procedures (0.3-0.5%) but significantly higher (up to 3.4%) in the context of acute coronary syndrome. Mortality associated with ST remains substantial, with 15-30% of patients succumbing to the event within 30 days [1].

In addition to the risk of thrombosis, ISR remains a prevalent clinical issue with a high potential for mortality and rehospitalization, despite decades of advancements in stent design and polymer coatings. Approximately 10% of patients experience ISR, with 25% of cases presenting as acute myocardial infarction (MI) [2]. These events carry a high 30-day mortality rate of 10-25% [2].

Our case involves a 63-year-old male with a history of hypertension and ischemic heart disease, who underwent PCI for proximal left anterior descending artery (LAD) and right coronary artery (RCA) lesions. During a five-and-a-half-year follow-up period, he developed three major stent-related complications: an episode of acute ST, a case of ISR, and a very late ST. These events required multiple interventional procedures and highlight the clinical complexity of managing long-term outcomes in patients with coronary stents.

## Case presentation

A 63-year-old male, former smoker, with hypertension under treatment, has been followed for ischemic heart disease since 2019 after an initial acute coronary syndrome treated with revascularization of the proximal LAD artery.

The initial presentation was an anterior ST-elevation myocardial infarction (STEMI). Coronary angiography revealed a thrombotic sub-occlusion of the proximal LAD involving the bifurcation with the first diagonal branch, and a significant proximal RCA stenosis. percutaneous coronary intervention (PCI) was performed on the LAD with thromboaspiration and direct implantation of a drug-eluting stent, without predilatation.

Four weeks after the index PCI, the patient was scheduled for a staged angioplasty to address a critical proximal RCA stenosis. At that time, he remained asymptomatic and was maintained on dual antiplatelet therapy (ticagrelor and aspirin), statins, antihypertensive treatment, and anti-ischemic therapy.

**Figure 1:**
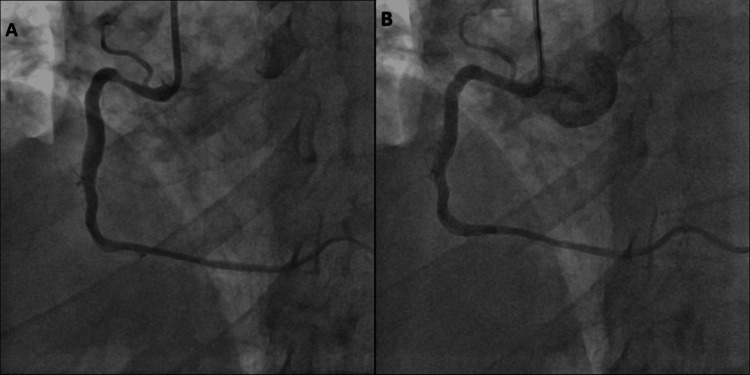
Coronary angiography images showing RCA stenosis and post-revascularization results. (A) Angiogram demonstrating significant stenosis in the proximal RCA prior to intervention. (B) Final angiographic result after drug-eluting stent implantation without predilatation, showing full vessel patency and no evidence of dissection. RCA: Right Coronary Artery.

Coronary angiography via the right radial artery (6F) showed no restenosis at the site of the previously implanted drug-eluting stent in the proximal LAD. A SYNERGY II (MR) 3.5 × 16 mm drug-eluting stent was successfully implanted in the proximal RCA without predilatation. The stent was deployed with a single inflation at 16 atm for 30 seconds. Final angiographic control confirmed the absence of dissection or thrombus, and Thrombolysis In Myocardial Infarction (TIMI) 3 flow was restored. As with the initial procedure, intravascular imaging modalities such as intravascular ultrasound (IVUS) or optical coherence tomography (OCT) were not utilized due to limited availability at our center at the time. Angiographic guidance was used for lesion assessment and stent optimization (Figure 1).

It is important to note that intravascular imaging techniques were not used during the initial procedure due to their limited availability at our center at that time, which may have influenced the detection of subtle procedural factors.

**Figure 2:**
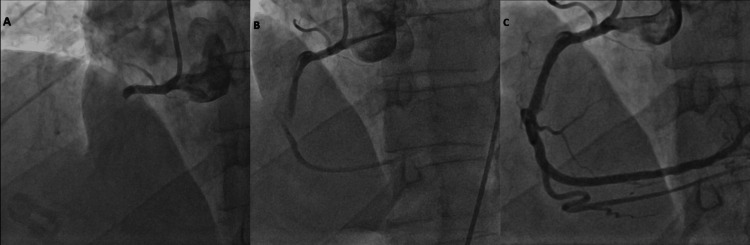
Angiographic images of acute stent thrombosis management at the distal edge of the newly implanted RCA stent. (A) Total occlusion at the distal edge of the RCA stent, caused by an edge dissection leading to thrombotic occlusion. (B) Result after thrombo-aspiration, showing partial restoration of flow. (C) Final angiographic outcome after implantation of a second drug-eluting stent overlapping the previous stent and post-dilatation with a non-compliant balloon, with TIMI 3 flow restored. RCA: Right Coronary Artery; TIMI: Thrombolysis In Myocardial Infarction.

One hour post-procedure, the patient developed sudden chest pain, with ECG showing ST-segment elevation in the inferior leads. Emergent repeat coronary angiography via the right femoral artery (6F) revealed an occlusion at the distal edge of the newly implanted stent due to dissection. This was managed with thromboaspiration followed by direct stenting with a drug-eluting stent, placed edge-to-edge with the previous stent, and post-dilatation using a non-compliant balloon. The intervention successfully restored TIMI 3 flow, alleviated chest pain, and resulted in resolution of ST-segment elevation in the inferior leads (Figure 2).

One year later, the patient presented with exertional angina, prompting a myocardial scintigraphy, which indicated ischemia involving the entire inferior wall, extending to the apical segment of the lateral wall. The patient was still taking dual antiplatelet therapy (ticagrelor and aspirin), statins, antihypertensive drugs, and beta-blockers.

**Figure 3:**
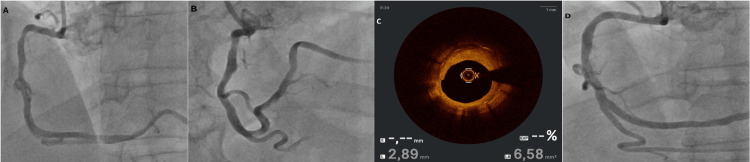
Coronary angiography and OCT of the RCA during in-stent restenosis assessment and treatment. (A, B) Angiographic images demonstrating an intermediate lesion at the superior bend of the RCA. (C) OCT imaging revealing approximately 60% in-stent restenosis within the previously implanted stent. (D) Final angiographic assessment after predilatation and drug-coated balloon angioplasty, showing a residual, non-significant lesion (<30%) with preserved TIMI 3 coronary flow. RCA: Right Coronary Artery; TIMI: Thrombolysis In Myocardial Infarction; OCT: Optical Coherence Tomography.

A coronary angiography was scheduled, revealing an intermediate lesion at the superior bend of the RCA. OCT was subsequently performed, identifying an in-stent restenosis of 60%. Based on these findings, we decided to proceed with angioplasty without stent implantation. The lesion was treated with predilatation using a non-compliant balloon, followed by drug-coated balloon angioplasty. Final angiographic assessment showed a residual, non-significant lesion (<30%) at the superior bend of the RCA. Coronary flow was normal (TIMI 3) (Figure 3).

Four and a half years after the previous event, a few days after discharge from the intensive care unit where he was hospitalized for septic shock secondary to orchiepididymitis complicating a prostate resection, the patient presented to the emergency department with anginal chest pain evolving over the past two hours.

Clinically, the patient was in shock, managed with vasoactive agents. ECG revealed ST-segment elevation in the inferior leads with extension to the right ventricle.

Transthoracic echocardiography demonstrated a mildly reduced left ventricular ejection fraction with impaired longitudinal function of the right ventricle.

At presentation, the patient was on single antiplatelet therapy with aspirin, along with statins, antihypertensive treatment, and beta-blockers, with no reported non-adherence to therapy.

**Figure 4:**
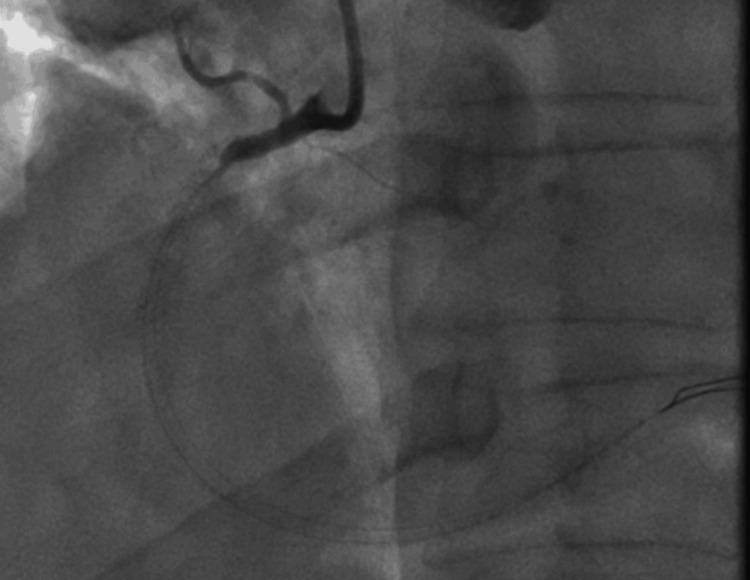
Coronary angiography illustrating very late stent thrombosis with total occlusion of the proximal RCA. Complete occlusion of the proximal RCA (Type C lesion >20 mm in length) at the site of the previously implanted drug-eluting stent, with no visualization of the distal vessel or antegrade flow. RCA: Right Coronary Artery.

Emergency coronary angiography via the right radial artery (6F) revealed an acute occlusion of the proximal RCA, exceeding 20 mm in length (Type C), originating from the site of the previously implanted drug-eluting stent. The occlusion extended into the mid-RCA, with no visualization of the distal vessel and absence of antegrade flow (Figure 4).

**Figure 5:**
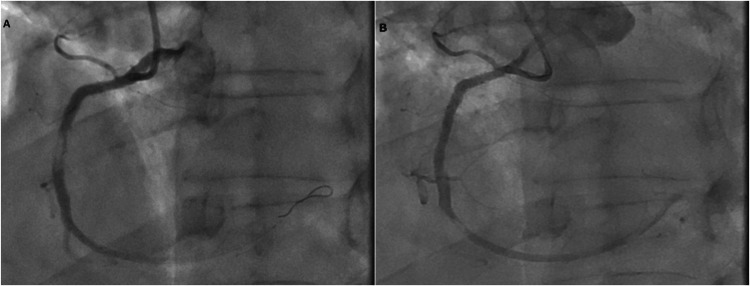
Angiographic images during the management of very late stent thrombosis showing intracoronary thrombus dynamics. (A) Floating thrombus observed in the mid-segment of the RCA following partial flow restoration after multiple thrombo-aspiration attempts and balloon dilations. (B) Distal migration of the intracoronary thrombus at the end of the procedure. RCA: Right Coronary Artery.

Management of the lesion was challenging, requiring thromboaspiration in combination with multiple balloon dilations using different balloon catheters. By the end of the procedure, a floating intracoronary thrombus persisted, with partial recovery of coronary flow (TIMI 2) (Figure 5).

At the end of the procedure, the patient was pain-free but remained with slight ST-segment elevation on ECG. The patient was initiated on tirofiban, unfractionated heparin, and dual antiplatelet therapy (aspirin and ticagrelor).

**Figure 6:**
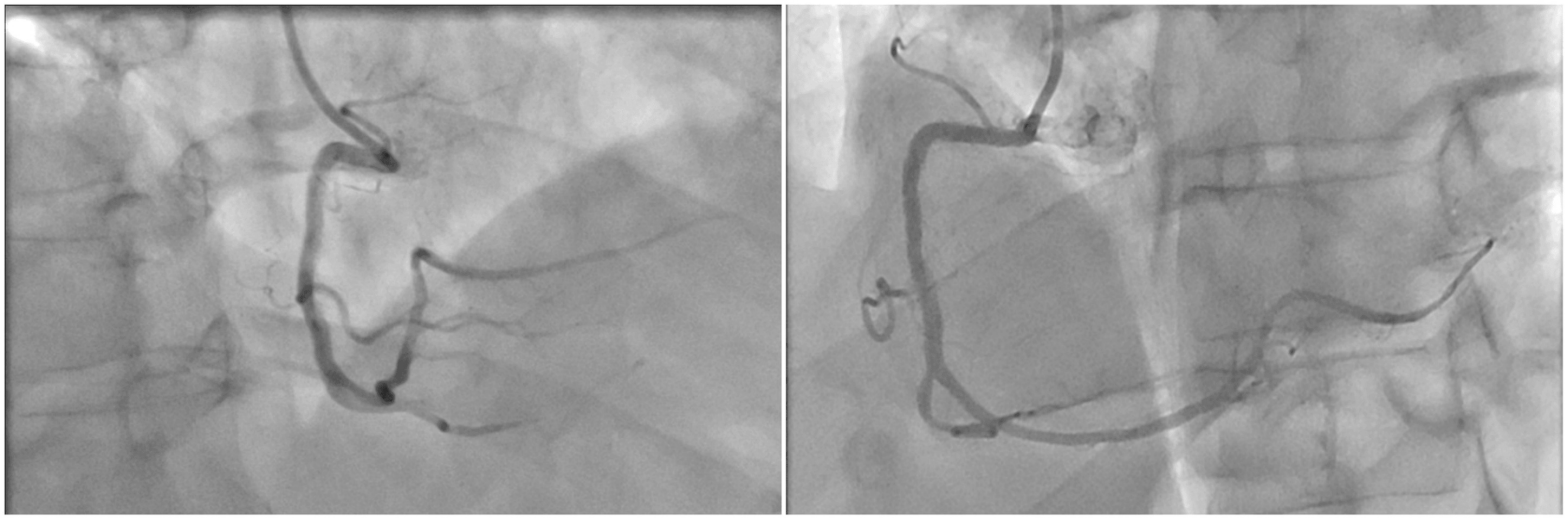
Follow-up coronary angiography images illustrating complete resolution of the intracoronary thrombus, restoration of normal RCA flow, and absence of residual stenosis. RCA: Right Coronary Artery.

A follow-up coronary angiography performed 48 hours later showed complete resolution of intracoronary thrombus images, with normal RCA flow and no residual stenosis (Figure 6).

The chronological sequence of interventions, diagnoses, and events over the 5.5-year follow-up period is summarized in Table 1.

**Table 1:** Chronological timeline of clinical events and interventions. A detailed overview of stent-related complications, interventional strategies, and clinical follow-up over a 5.5-year period in a patient with ischemic heart disease. ACS: Acute Coronary Syndrome; PCI: Percutaneous Coronary Intervention; LAD: Left Anterior Descending artery; DES: Drug-Eluting Stent; RCA: Right Coronary Artery; ISR: In-Stent Restenosis; DCB: Drug-Coated Balloon; NC balloon: Non-Compliant Balloon; STEMI: ST-Elevation Myocardial Infarction; LV: Left Ventricle; RV: Right Ventricle; TIMI: Thrombolysis In Myocardial Infarction; UFH: Unfractionated Heparin; DAPT: Dual Antiplatelet Therapy.

Date / Timeframe	Clinical Event / Intervention
2019	Initial diagnosis of ischemic heart disease after an acute coronary syndrome (ACS); PCI of proximal LAD with DES.
+ 4 weeks	Scheduled staged PCI of proximal RCA; DES (SYNERGY II 3.5 × 16 mm) implanted without predilatation.
1 hour later	Acute stent thrombosis due to distal edge dissection; managed with thromboaspiration and stenting (SYNERGY II 2.75 × 38 mm); post-dilatation with non-compliant balloon.
+ 1 year	Angina on exertion; stress myocardial perfusion imaging showed inferior wall ischemia. Coronary angiography: 60% ISR at superior RCA bend. Treated with DCB angioplasty (AGENT 3.5 × 20 mm) after predilatation with NC balloon.
+ 4.5 years	Hospitalized for septic shock due to post-op orchiepididymitis.
Few days post-discharge	Presented with inferior STEMI and cardiogenic shock; ECG: ST elevation in inferior and right leads; echocardiography: mild LV dysfunction with RV involvement. Coronary angiography: very late stent thrombosis of proximal RCA stent (Type C occlusion >20 mm); thromboaspiration and balloon dilation. Residual floating intracoronary thrombus. Treated with tirofiban, UFH, and DAPT; TIMI 2 flow post-procedure.
48 hours later	Repeat angiography: complete thrombus resolution, TIMI 3 flow, no residual stenosis.

## Discussion

In 2006, the Academic Research Consortium established universal definitions for ST, categorizing it based on the timing of occurrence and the level of diagnostic certainty, namely as definite, probable, or possible.

ST is classified by timing as follows: acute ST occurs within the first 24 hours following stent implantation; subacute ST occurs between 24 hours and 30 days post-implantation; late ST occurs between 30 days and one year; and very late ST is defined as occurring beyond one year after the procedure [3].

The diagnosis of definite ST requires the identification of a thrombus within the stent or within a 5 mm segment proximal or distal to the stent, accompanied by at least one of the following clinical criteria within 48 hours: acute onset of ischemic symptoms at rest, new ischemic electrocardiographic changes indicative of acute ischemia, or a characteristic rise and fall in cardiac biomarkers [3].

Numerous clinical and technical risk factors contribute to ST, which can be categorized into clinical, procedure-related, lesion-related, stent-related, and antiplatelet-related factors.

Procedural risk factors are the most critical determinants of early ST. Key predictors include stent undersizing, residual dissection, impaired TIMI flow, and residual disease [2,4]. Additionally, patient-specific factors such as reduced left ventricular function and impaired responsiveness to adenosine diphosphate (ADP)-antagonist therapy further elevate the risk [2,4]. On the other hand, very late ST is primarily driven by factors such as stent malapposition, uncovered struts, neoatherosclerosis, and stent underexpansion [2,4]. 

ISR is defined angiographically as a narrowing exceeding 50% of the vessel diameter within the stented segment [2,5]. Clinically relevant ISR is determined by combining the assessment of luminal narrowing with the patient’s clinical context, which may include recurrent angina or objective signs of ischemia. For intermediate lesions, fractional flow reserve or intravascular imaging techniques can aid in guiding clinical decisions [2,5].

Coronary angiography remains the gold standard for diagnosing and classifying ISR. The SCAI classification integrates the timing of ISR onset with its underlying mechanism to aid diagnosis and treatment. It defines three timing categories: early ISR (within 30 days), typically resulting from stent undersizing, underexpansion, or fracture; late ISR (30 days to 1 year), often associated with delayed healing, uncovered stent struts, or intimal hyperplasia, particularly in bare metal stents (BMS); and very late ISR (after 1 year), frequently linked to neoatherosclerosis, intimal hyperplasia, or stent fracture [2]. 

Treatment of ST begins with diagnostic angiography, followed by initial management with balloon angioplasty, sometimes accompanied by thrombus aspiration in cases of high clot burden [2]. Additional stenting is generally reserved for significant residual dissections, particularly if recent dual antiplatelet therapy has been interrupted [2]. Ensuring optimal stent apposition through high-pressure inflations with non-compliant balloons is crucial. Glycoprotein IIb/IIIa antagonists may aid in improving microvascular reperfusion, while prolonged anticoagulation and enhanced antiplatelet therapy should be considered in cases of residual thrombus or drug resistance [2]. Mechanical thrombectomy remains investigational due to a lack of large-scale studies [2]. Ultimately, thorough post-procedure optimization and addressing the underlying cause of ST are essential for improving patient outcomes and preventing recurrence.

The treatment of ISR is multifaceted, with the optimal approach tailored to the underlying mechanism and patient characteristics. Medical therapy, including aspirin and statins, follows guideline recommendations, though no pharmacologic agent has proven effective in halting ISR progression [6]. Balloon angioplasty remains a primary option, particularly for focal lesions, though it carries a high recurrence risk [6]. Cutting and scoring balloons aid in lesion preparation but lack definitive evidence for clinical benefit [6]. Vascular brachytherapy was historically used for ISR in BMS but has largely been replaced by DES, which have demonstrated superior outcomes [6]. Rotational atherectomy and laser techniques are adjunctive tools for heavily calcified lesions but are not standard treatments [6]. Repeat stenting with DES is a widely accepted strategy, reducing restenosis rates,v particularly with second-generation stents, though recurrent ISR remains a challenge [6]. DCB provide an alternative by delivering antiproliferative agents without additional stent layering, showing promise in both bare-metal and drug-eluting ISR [6]. Bioresorbable vascular stents offer temporary support while delivering anti-proliferative drugs, but their outcomes remain inferior to second-generation DES [6]. Among these options, DES and DCB are the most widely used, with ongoing research focused on improving long-term outcomes and reducing recurrence [6].

Our patient initially presented with an anterior STEMI and was found to have two-vessel disease: a thrombotic sub-occlusion of the proximal LAD extending to the diagonal (culprit lesion), and a significant proximal RCA stenosis. He underwent urgent PCI of the LAD, which was treated with DES implantation without predilation, guided by angiography alone. Four weeks later, a staged PCI of the RCA was performed, also without predilation, using a 3.5 mm × 16 mm SYNERGY II stent with high-pressure post-dilation at 16 atm for 30 seconds.

No IVUS or OCT was used in either procedure, as these modalities were unavailable at our center. The decision not to predilate was made based on angiographic assessment and good guide catheter support.

Our patient experienced two episodes of stent thrombosis and one episode of in-stent restenosis over the follow-up period. The acute stent thrombosis occurred as a result of residual dissection following the initial stent implantation. The very late stent thrombosis was likely precipitated by septic shock in the setting of a residual lesion that remained after DCB angioplasty for previously diagnosed in-stent restenosis. The late ISR was associated with a combination of patient-related risk factors and procedural factors, including a prior dissection and likely stent underexpansion.

Importantly, systemic inflammation, in the form of septic shock, likely contributed to the pathogenesis of the very late thrombosis, reflecting how systemic triggers may exacerbate endothelial dysfunction and pro-thrombotic states even years after stent implantation.

For each complication, the patient was managed with a tailored therapeutic strategy based on the specific clinical context and the available interventional tools. In all instances, the interventions resulted in satisfactory clinical and angiographic outcomes.

## Conclusions

ST and ISR remain critical challenges in interventional cardiology, necessitating individualized treatment strategies. This case underscores the complexity of managing recurrent stent-related complications over time. Ongoing advancements in imaging and therapeutic modalities, such as DCB and next-generation stent technologies, continue to enhance the management of ISR and ST. Future research should aim to optimize long-term outcomes and reduce recurrence through improved stent design and more effective antithrombotic strategies.
